# Forest malaria and prospects for anti-malarial chemoprophylaxis among forest goers: findings from a qualitative study in Thailand

**DOI:** 10.1186/s12936-022-04070-4

**Published:** 2022-02-14

**Authors:** Monnaphat Jongdeepaisal, Panarasri Khonputsa, Orathai Prasert, Suphitsara Maneenet, Kulchada Pongsoipetch, Anchalee Jatapai, Chawarat Rotejanaprasert, Prayuth Sudathip, Richard J. Maude, Christopher Pell

**Affiliations:** 1 Mahidol-Oxford Tropical Medicine Research Unit, Faculty of Tropical Medicine, Mahidol University, Bangkok, Thailand; 2 Centre for Tropical Medicine and Global Health, Nuffield Department of Medicine, University of Oxford, Oxford, UK; 3 Department of Tropical Hygiene, Faculty of Tropical Medicine, Mahidol University, Bangkok, Thailand; 4 Division of Vector Borne Diseases, Department of Disease Control, Ministry of Public Health, Nonthaburi, Thailand; 5 Harvard TH Chan School of Public Health, Harvard University, Boston, USA; 6 The Open University, Milton Keynes, UK; 7 Amsterdam Institute for Global Health and Development (AIGHD), Amsterdam, The Netherlands; 8 Department of Global Health, Amsterdam University Medical Centers, Amsterdam, The Netherlands; 9 Centre for Social Science and Global Health, University of Amsterdam, Amsterdam, The Netherlands

**Keywords:** Forest goer, Malaria, forest, Malaria, intervention, Malaria, prophylaxis

## Abstract

**Background:**

Across the Greater Mekong Subregion, malaria remains a dangerous infectious disease, particularly for people who visit forested areas where residual transmission continues. Because vector control measures offer incomplete protection to forest goers, chemoprophylaxis has been suggested as a potential supplementary measure for malaria prevention and control. To implement prophylaxis effectively, additional information is needed to understand forest goers’ activities and their willingness to use malaria prevention measures, including prophylaxis, and how it could be delivered in communities. Drawing on in-depth interviews with forest goers and stakeholders, this article examines the potential acceptability and implementation challenges of malaria prophylaxis for forest goers in northeast Thailand.

**Methods:**

In-depth interviews were conducted with forest goers (n = 11) and stakeholders (n = 16) including healthcare workers, community leaders, and policymakers. Interviews were audio-recorded, transcribed and coded using NVivo, employing an inductive and deductive approach, for thematic analysis.

**Results:**

Forest goers were well aware of their (elevated) malaria risk and reported seeking care for malaria from local health care providers. Forest goers and community members have a close relationship with the forest but are not a homogenous group: their place and time-at-risk varied according to their activities and length of stay in the forest. Among stakeholders, the choice and cost of anti-malarial prophylactic regimen—its efficacy, length and complexity, number of tablets, potential side effects, and long-term impact on users—were key considerations for its feasibility. They also expressed concern about adherence to the preventive therapy and potential difficulty treating malaria patients with the same regimen. Prophylaxis was considered a low priority in areas with perceived accessible health system and approaching malaria elimination.

**Conclusions:**

In the context of multi-drug resistance, there are several considerations for implementing malaria prophylaxis: the need to target forest goers who are at-risk with a clear period of exposure, to ensure continued use of vector control measures and adherence to prophylactic anti-malarials, and to adopt an evidence-based approach to determine an appropriate regimen. Beyond addressing current intervention challenges and managing malaria incidence in low-transmission setting, it is crucial to keep malaria services available and accessible at the village level especially in areas home to highly mobile populations.

**Supplementary Information:**

The online version contains supplementary material available at 10.1186/s12936-022-04070-4.

## Background

Across the Greater Mekong Sub-region (GMS), malaria incidence and mortality have reduced significantly over the past 20 years [1]. In part, this success is a result of national malaria control programmes (NMCPs) that have improved the availability of effective anti-malarial treatment, access to early diagnosis, and coverage of prevention measures [2]. As incidence has declined across the GMS, and to address the emergence and spread of anti-malarial resistance—including to artemisinin-based combination therapy (ACT) [3]—countries have set ambitious goals to eliminate malaria by 2030 [1]. However, malaria remains a dangerous infectious disease in the GMS, particularly for migrants and people who visit forested areas—often along international borders—where residual transmission continues.

To accelerate malaria elimination, Thailand’s national malaria programme has been putting in efforts to sustain existing malaria services, including provision of testing, treatment, and prevention in endemic areas [4]. Case surveillance has resulted in a substantial reduction in the number of active malaria foci [5] and decline in malaria burden [6]. Although vector-control measures have been introduced in endemic communities, outdoor biting vectors remain a key challenge to efforts to protect this population group who mostly engage in night-time long working hours in the forest and forest fringes [7]. A recent foci cohort analysis described the challenges of malaria control in border regions with highly mobile migrant workers (Prachinburi) and high numbers of military personnel (Yasothon), and in areas experiencing political and social unrest (endemic areas in Yala) [8]. A recent systematic review highlighted the limited protection that vector control and village-based measures offer forest goers and the need for a more tailored package of interventions [9].

Anti-malarials have been used to prevent malaria infection and lessen its severity [10, 11] among different at-risk populations in varied contexts. To accelerate elimination, mass drug administration (MDA) has recently been implemented in areas where transmission is low [12–14]. Seasonal malaria chemoprevention (SMC) has been used in seasonal-transmission settings [15] and intermittent preventive treatment (IPT) [16] has been administered to pregnant women [17], infants and children in endemic areas [18, 19]. Chemoprophylaxis is often provided to travellers and military personnel visiting transmission areas [20]. Several studies have recorded the use of malaria prophylaxis in Thailand in the past: a trial with migrant workers in eastern rural areas [21], and Thai soldiers along the Thai-Cambodia border (1987–1991) [22–25].

Malaria prophylaxis has, however, not generally been recommended for indigenous population living in endemic areas. In light of limited effective vector control strategies and determined periods of exposure during forest visits, it has been proposed as a strategy to protect forest goers [9, 26–28] and is currently being trialled in Cambodia [29]. To maximize the impact of this approach, additional information is needed to understand activities of forest goers and their willingness to use different prevention measures, including chemoprophylaxis, and how it could be delivered in their communities.

Drawing on in-depth interviews, this article explores the experiences with malaria of forest goers and the perceptions of community stakeholders and policymakers on forest malaria prevention and control. The aim is to develop recommendations for future implementation of prophylaxis as part of malaria control programmes in Thailand and the wider GMS. The article characterizes forest going activities, the livelihoods and experiences of forest goers with malaria prevention and health services in endemic communities. Interviews with healthcare workers, community leaders, and policymakers sought to identify and describe their perspectives on malaria intervention implementation and challenges, including the feasibility of malaria prophylaxis and the evidence needed for decision-making about its possible implementation.

## Methods

### Setting

**Fig. 1:**
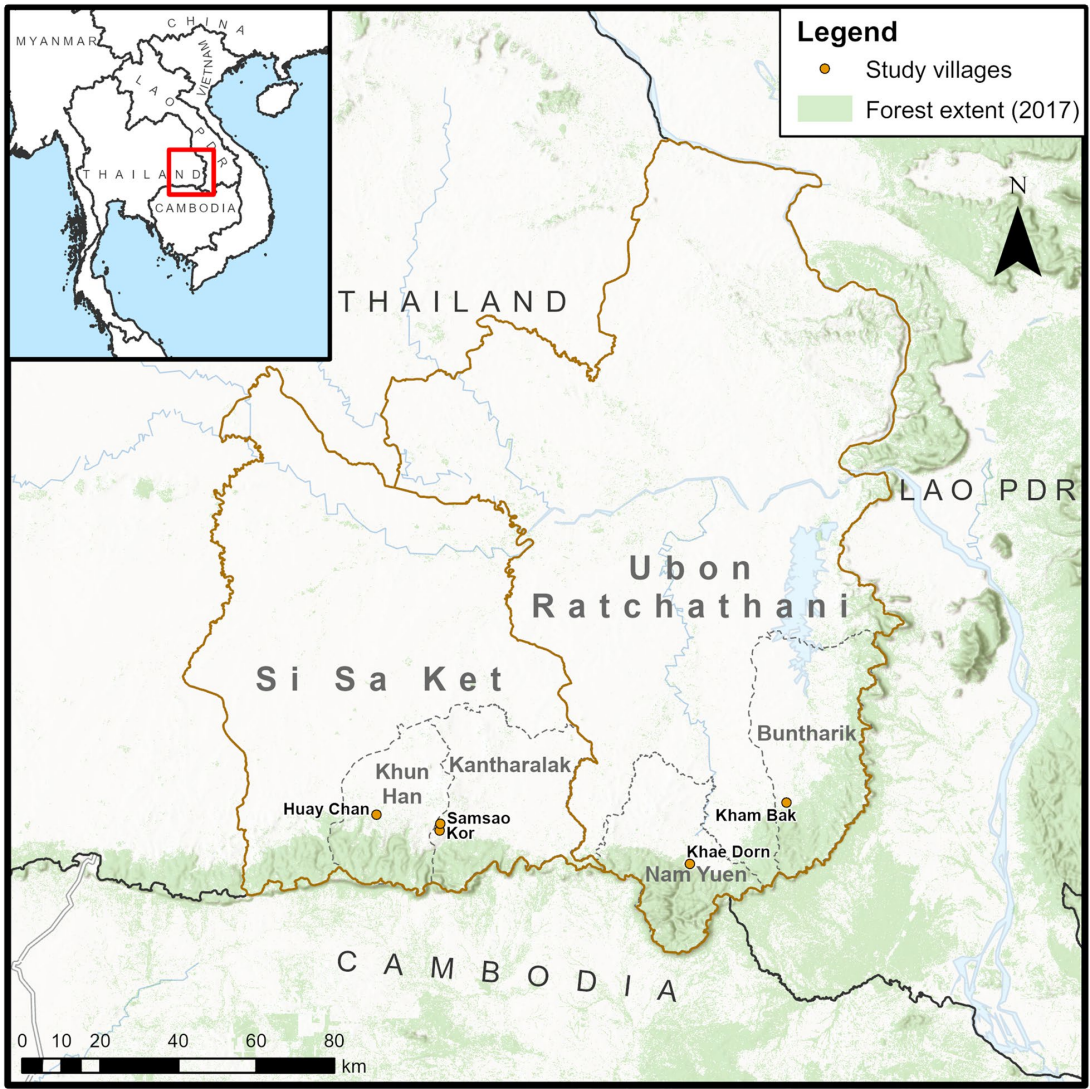
Map of study villages and districts. Uses Esri World Hillshade [basemap] and World Terrain Base [basemap] accessed on 6 September 2021

The study was conducted in four malaria endemic villages in Ubon Ratchathani (UB) and Si Sa Ket (SSK) provinces bordering Lao PDR and Cambodia (Fig. 1). The majority of community members work on agricultural land, particularly rubber farms and rice fields, with farming their main source of income. In the villages, selected local residents—Village Malaria Workers (VMWs), also referred to as Malaria Post workers (MPWs)—provide village-based malaria services, such as diagnosis with a rapid diagnostic test (RDT), anti-malarial treatment, and distribution of insecticide-treated nets (ITNs) [30]. The Malaria Clinic (MC) and district hospital are other points of care for community members, usually within 10–20 km of the villages. The VMWs are mainly supervised by the Provincial Health Department, and work with community leaders, MC staff, and local non-governmental organization to provide malaria services in the villages and border areas.

### Ethical considerations

The study was approved by the Oxford Tropical Research Ethics Committee (OxTREC reference no. 534-19) and the Ethics Committee of the Faculty of Tropical Medicine, Mahidol University (TMEC 20-012). All respondents provided written informed consent to participate in the study and for the interviews to be audio-recorded. Consent was obtained from villagers when they were recruited, in their village or at their home. For stakeholders, written informed consent was obtained before the interviews at their places of work. Local community members and the Division of Vector Borne Diseases (DVBD) staff were engaged in the study from an early stage. Before beginning data collection, meetings with community leaders and healthcare workers were held to explain about the study and its purpose. The study team recorded their observations of the stakeholder and community meetings and took notes of their opinions and questions to understand the local context and inform the interview approach.

### Data collection

In-depth interviews were conducted with forest goers who reported visiting forested areas on more than 14 days/year and were aged 18 years or older. Respondents were selected based on a mixture of purposive and snowballing approaches. With assistance of staff at local healthcare facilities, VMWs and village leaders were identified from their roles in the communities. VMWrs and leaders subsequently assisted with identifying initial groups of community members who made regular forest trips or had experience of malaria. Respondents were approached and interviewed at their homes or communal places in the communities. The interviews were conducted during October 2020–January 2021 by trained field researchers fluent in Thai and Isaan (northeastern Thai), the local languages in the communities. The interviews took 45–60 min on average and respondents were compensated for their time.

Consenting respondents were asked about forest-related activities, experience with malaria, use of prevention measures, and their perception of prophylaxis. Additional in-depth interviews were conducted with local healthcare workers, community leaders, and policymakers about current and future approaches to address forest malaria and implementation of malaria interventions including prophylaxis. Regular debrief meetings were conducted among the study team and field researchers throughout the study. The total number of interviews was determined by a point of saturation whereby no further novel information was forthcoming from subsequent data collected.

In-depth interview (IDI) guides for each type of respondent were developed based on the initial topics drawn from a recent qualitative study on forest going and malaria-related risk in Cambodia [27]. IDI guides for each group of interviewees (see Additional files 1, 2, 3, 4) were initially designed in English and translated into Thai by a native Thai speaker and researcher. The guides included key topic areas and a list of suggested questions, and were designed to be used in a flexible and iterative manner: interviewers would be reactive to the responses and probe or ask follow-up questions to elicit the information on specific topics emerging during the interviews. During development, the translation of the topic areas and suggested questions were discussed and checked with the team who were also trained on how to use the guides. The guides were then piloted with the first recruited study respondents to check for any miscommunication and revised as necessary.

### Data processing and analysis

After respondents gave their consent, interviews were audio-recorded and subsequently transcribed by field researchers and translated to English by a bilingual social scientist. The translated transcripts and detailed interview notes were imported into NVivo version 12 (QSR International Australia) for qualitative thematic analysis. All transcripts were read several times and coded line-by-line using inductive and deductive approaches [31]: the codebook used was initially based on the main research topics. Subsequently, during the process of coding, themes that emerged from the data were incorporated into the codebook. The map in Fig. 1 was created using ArcGIS Pro software version 2.5.0 (Esri, Redlands, CA). National and provincial administrative boundaries from Global Administrative Areas version 3.6 (https://gadm.org/download_country_v3.html), district-level administrative boundaries from the Thailand Subnational Administrative Boundaries dataset 2019, Royal Thai Survey Department, and forest extent 2017 from Global Forest Change 2000–2020 (https://earthenginepartners.appspot.com/science-2013-global-forest).

## Results

### Demographic characteristics of respondents

The findings presented are based on individual in-depth interviews with 11 forest goers: they are male adult community members who were more engaged in multiple forest activities and thus at a higher risk for malaria than female members in the community. These at-risk populations were also reflected in previous studies in Thailand [27, 32]. A further 16 interviews were conducted with healthcare workers, local community leaders, and policymakers at provincial and national levels. Characteristics of the respondents are summarized in Table 1.

**Table 1:** Demographic characteristics of respondents

Forest goers	
Sex	
Female	0
Male	11
Age	
31–40	2
41–50	4
51–60	5
Marital status	
Single	1
Married	9
Not specified	1
Number of household members	
1–2	2
3–4	9
Education years	
1–6	7
7–12	4
Stakeholders	
Sex	
Female	8
Male	8
Age	
31–40	4
41–50	6
51–60	6
Occupation	
Healthcare workers	8
Community leaders	4
Policymakers	4

### Forest visits

Forest visits were described as key to community members’ livelihoods. Forest goers reported earning money from collecting and selling a wide range of wild products. The forest was seen as their “backyards” or “market”, where villagers grow and collect food for their family or for sale. Collecting wild products was a year-round forest activity and community members collected different goods in different seasons: mushrooms in the wet season; beeswax and honey in the dry season; and various types of vegetable in any month of the year. For these activities, respondents described how male and female adults would visit the forest and come back to sell the products the same day. Two respondents also mentioned recreational forest visits to waterfalls or streams with their families.

**Fig. 2:**
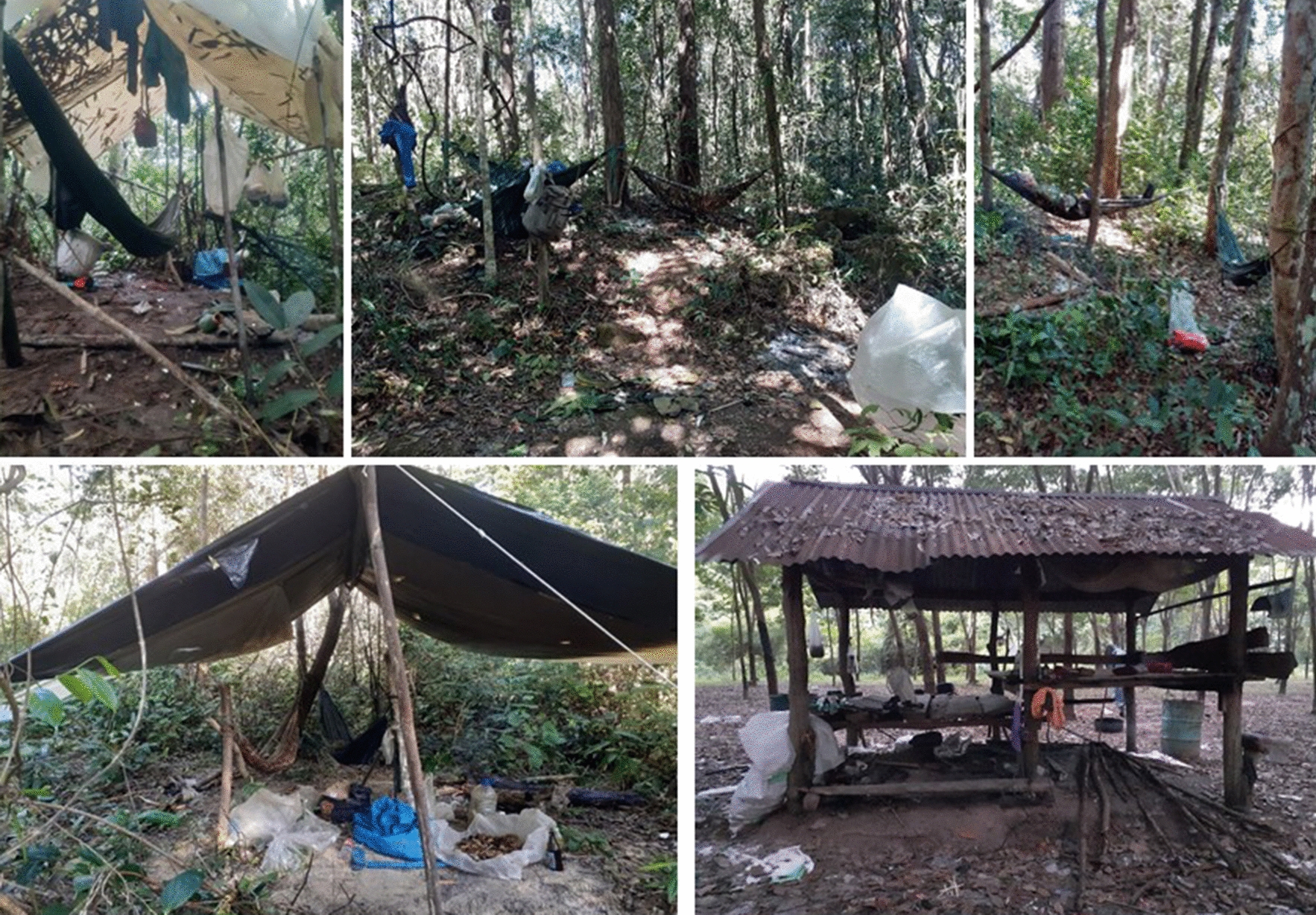
Sleeping arrangements in the forest and forest farm **A**–**D**. Make-shift sleeping arrangement in the forest with and without hammocks and **E** outdoor farmhouse in a rubber plantation for resting

Several rubber farms and plantations were located along the forested area near to the villages, and were often referred to as forest farms or rubber forest where many community members made their living. Respondents described long working hours in the rubber farms from night-time until early morning from 12 a.m. to 7 a.m. Male and female members of the family, occasionally with children, would spend nights at their farmhouse, a small hut usually without electricity (Fig. 2). A cycle of rubber farm work was described as two continuous days with one rest day interval, during which respondents often foraged in the forest close to their farms. Other crops such as rice, cassava, and cashew nuts were also common agricultural yields and the main source of income for most villagers in the areas, however they were normally not grown in or near to the forest.

Rainy weather, busy work in the rubber farm and rice fields, and stricter enforcement of logging and hunting bans led to fewer overnight forest visits. Forest visits were more frequent after harvesting or during the dry season from December to February; many also described that travelling and finding a sleeping place in the forest during this period was more convenient (less rain) and thus preferred. A few mentioned visiting the forest more frequently in the rainy season to supplement their lost income from rubber work, during which they could not extract the rubber as much. Due to recent falls in the price of rubber, some respondents mentioned that they often made additional income from selling forest products and farming different types of crops.

Apart from local residents, temporary workers also engaged in agricultural work in the villages, especially in rubber plantations, and occasionally visited the forest during their stay in the village. Respondents described how those workers included people from other provinces and local residents who did not own, or had previously sold, their land. Non-residential workers would stay and work in the farms during the rubber season and return home after the work was done.

For some, forest visits were seen as a risky undertaking due to the authorities’ stringent enforcement of logging and hunting bans in the conservation areas, where forest rangers were identified as “invading” the forest. Hunting animals, which was mostly undertaken by male forest goers, could yield as much as a week’s income for their families from a single hunt. Respondents reported infrequent hunting trips in recent times, describing avoiding hunting in restricted areas when officials were on patrol for fear of being detained or having their hunted animals confiscated. Logging Pa-Yung trees or rosewood in the areas was described as widespread in the past but less common among Thai and Khmer villagers and non-residential workers compared to recently during 2016–2017 in the mountainous and forested areas bordering Cambodia.

The boundaries between forested areas and village settlements or farms were often described as unclear, especially for those living or farming on the fringe or edge of the village and forest. Some of these forest fringes were identified as protected areas where logging, hunting, and occasionally farming was restricted. Some respondents described that they could still live and work on their own land, whereas others reported having to relocate or being unable to earn a living from such land. The risk of getting caught in the restricted areas discouraged some respondents from farming or foraging in the forest.

In addition to forest goers and villagers, Buddhist monks residing in temples at the edge of the village (also referred to locally as “forest temples”) also made occasional trips to the forest. Respondents described how local residents and visitors often visited and stayed at the temples, particularly for rituals during the Buddhist Lent months in the rainy season. Conservation authorities (or “forest staff”) and military scouts were also said to patrol the area from camps in and around the national parks.

### Experience of malaria prevention, testing and treatment

#### Perceived risk of malaria

Respondents described malaria risk associated with forest visits and contact with mosquitoes in general. Many reported being bitten by mosquitoes in the forest, particularly during working in hot and humid weather. Some also described that a person who is unhealthy or has a “weak body” from working hard in the farms is more prone to get malaria. Forest goers referred to hotspots in the forest, including caves and cave-like locations and areas near to water sources. They reported getting many mosquito bites at these places but described them as convenient locations to make fire, cook, wash, and rest when it rained. Some respondents also reported nuisance from mosquito bites during work in the rubber farm at night, and in their hut or farmhouse.

"*Mostly locals visit the forest to find wild products. It’s their way of life, to make use of the forest, such as foraging and picking mushroom. Some might not be aware of malaria risk from mosquito bites … Many in the village were also hired to work in rubber farms at night, so they are also at risk of mosquito bites."*

IDI with village leader from Samsao village, SSK.

#### Use of multiple prevention measures

Forest goers were well-aware of how to protect themselves from malaria in the forest. Making fire, wearing long-sleeved clothes, using mosquito repellent and coils, and sleeping under mosquito nets were mentioned. Some respondents also described bringing a hammock net to the forest. Their use of protective measures varied across forest settings and activities. Most respondents described wearing long-sleeved clothes and other accessories, such as gloves, boots, and balaclava as necessary for their forest visit. A few respondents preferred not to use repellents or coils when they were hunting or tracking animals, describing the scent of the chemicals as potentially revealing their whereabouts or creating unwanted attention. One respondent described avoiding making fire in the forest, which was usually used to keep mosquitoes away or to hunt bees, for fear of causing a wildfire and getting arrested. During these trips, mosquito nets are sometimes perceived to be less prioritized among forest goers when their forest tasks required packing heavy necessities and valuable products.

*"If I brought a mosquito net with me, I slept under it, but sometimes I forgot. The net is impregnated but I usually forgot to bring it with me because there are so many things, food and other supplies … very heavy. If I found an animal I would leave my things in the forest. Next time I go, everything is there already in the forest, the rice and pots. I left them at my regular spots."*

IDI with male, 53-year-old forest goer from Kor village, SSK.

For rubber plantation workers, mosquito coils were preferred and carried around the plantation during work at night. Workers would use 2–3 coils for 5–6 h of work in the plantation and around their farmhouse. The equipment was described as fairly accessible to forest goers: respondents reported purchasing staples, such as coils and repellent themselves from local shops, and receiving mosquito nets including LLINs and LLIHNs from VMWs in their villages and Malaria Clinic staff. Two forest goers reported other types of prevention they or other members of the community had used, for example, a herbal drink from tree roots or taking contraceptives.

#### Experience of malaria symptoms, testing and treatment

Forest goers reported having had malaria before: “too many times” in some cases. When asked specifically how many times, responses included: “10–15 times” and “more than once a year”. Others referred to one or two bouts in total. Respondents who had had many bouts of malaria reported being able to recognize malaria based on past experience of symptoms. Many described symptoms after returning from the forest. Convulsion, unconsciousness, chills and high fever were seen as severe symptoms and cycling fever was mentioned as the main feature to distinguish malaria from other febrile illnesses.

Respondents described being able to distinguish between different types of malaria from their symptoms. Some were aware from prior experience of being diagnosed by healthcare workers. A few recognized the treatment doses, correctly describing 14 days of treatment for *Plasmodium vivax* and a shorter regimen for *Plasmodium falciparum* (3 days for most regimens). Respondents reported getting better after the treatment and attending for follow-up. A few were aware that a malaria patient should take the treatment drugs as prescribed and not miss doses, explaining that the patient may get sick again if s/he did not take the tablets as prescribed.

When they suspected malaria, forest goers described visiting several options of care providers: VMW or MPW in the village, MC or hospitals in the district. The VMWs or local MPW were the preferred option for malaria diagnosis using RDT: the village-based service was described as fast and specific to malaria, allowing respondents to go quickly back home or to work. Local healthcare workers described how the local health centre or sub-district hospital staff usually referred patients to VMWs for a malaria test if they were suspected of having malaria. VMWs would often advise the patient to return and re-test if s/he had a negative result but had ongoing symptoms.

Some respondents reported making the longer trip to a MC and/or district hospital because they preferred help from a medical doctor. A few described disliking the district hospital because of the time burden, mainly because they might be hospitalized for a few nights. A healthcare worker explained how some patients also refused to visit district hospitals because they do not want to disclose personal information such as their travel history. Some were said to be concerned about medical tests there, which may reveal their use of illegal substances. Respondents reported visiting the closest hospital to their village. There were mentions of forest goers purchasing common medicine to treat fever from a local pharmacy or visiting a private clinic because of their long opening hours and fast service.

### Addressing forest malaria and implementing malaria interventions

#### Provision of malaria services

Stakeholder respondents described several challenges for the provision of malaria services. There was a lack of staff trained to diagnose malaria with microscopy at district level, which had resulted from the retirement of senior staff, lack of incentive for trained staff to relocate from the provincial unit, and the absence of training for the current staff. Respondents highlighted that this skill is important to confirm parasite levels during diagnosis or follow-up, and reported having to ship samples (thick and thin smears) to provincial staff for microscopy tests. Provincial staff also expressed concern about lower capacity of RDTs to detect malaria and how the test kits should be properly maintained at the VMW’s home. 

Healthcare workers reported that implementing certain services, such as distribution of LLINs and LLIHNs by a sub-district hospital might be slow. They highlighted the provision of malaria care and information by VMWs as crucial to reach forest goers, comparing this to a dengue programme for which patients otherwise visited the district hospital for care (dengue is also prevalent in the study areas). One national programme member suggested that integrating the malaria programme into the existing local health system, similar to the dengue programme which has been under the sub-district health fund to address communicable disease, could help to sustain the programme. The respondent explained that the role of national staff should then be to design interventions and conduct monitoring and evaluation of malaria programmes.

#### Case surveillance

Local malaria staff outlined the challenges of implementing case surveillance and targeting officials who also regularly visit the forest. Respondents expressed concern about possible ongoing malaria transmission among military and forest officials, and reported difficulties in reaching this population group due to restrictions on visiting the areas. The military-restricted area was described as a barrier to implement Day-7 of the 1-3-7 strategy [6]: staff were not able to perform focus investigations and implement vector-control measures, such as indoor residual spraying (IRS). They suggested that the intervention should also engage and provide malaria services to the officials at their camps in order to conduct case investigations and subsequently foci investigations in the endemic area. Respondents also reported that the officials usually went directly to district hospitals which provided care of the army officials or they might be treated by medical personnel within the unit itself.

#### Low adherence to malaria treatment

National malaria programme staff related concerns about low adherence to malaria treatment among *P. vivax* cases due to the 14-day regimen length, particularly in areas with high cross-border mobility, giving the examples of Mae Sot in Tak bordering Myanmar, and Yala bordering Malaysia. Local health care workers described how patients may be discouraged from attending a follow-up visit in the district because they did not want to spend time away from home or from work. Some also explained that their trust in, and relationship with, the providers are important to encourage patients to attend follow-up at a health facility. In addition, policymakers described that malaria is perceived as primarily a febrile illness, with patients likely to cease a treatment regimen when symptoms ease. This was compared to tuberculosis or HIV patients whose “burden” was perceived to be heavier due to the longer duration of symptoms and social stigma of the diseases. The respondent also pointed out that malaria treatment in the form of tablets or pills may be perceived as “western medicine” that can accumulate and negatively affect a person’s health long-term. Patients may thus avoid taking many pills when they already feel better or consider it unnecessary.

*“Many people asked why patients do not take all medicine as prescribed. From my experience, those with colds rarely adhere to the prescription, sometimes they take all and sometimes they don’t…it is quite natural. If we compare this with tuberculosis or HIV, it is different because when malaria patients are treated they feel better, no fever. But for TB patients they suffer from exhaustion, difficulty breathing, problems with their lungs.*

*[…] There is a belief that western medicine has many side effects which may accumulate when taking it for a long period of time. It’s their belief but people overlook that these medicines, the tablets that we took…, a whole lot of research has gone into producing each one so that we know exactly the proportion of their effect on people. We are aware of their side effects.”*

IDI with national policymaker.

A policymaker respondent also outlined the challenge of training district hospital staff on new and updated malaria guidelines. The respondent explained how medical staff often treated patients based on their empirical experience, and felt that local staff were more familiar with older anti-malarial regimens, such as quinine, and perceived them to be better (more efficacious) for treatment. Respondents described the need to explain how to administer new treatment regimens, such as artesunate, and why it is important to change to prevent the spread of drug resistance.

#### Approaches to malaria prevention and control

To address these challenges to malaria control, several strategies were identified. National malaria programme staff outlined how a new treatment regimen, namely tafenoquine, and quantitative G6PD testing, were needed to address low adherence among *P. vivax* cases and to ensure proper prescription of the regimen. For malaria diagnosis, use of high sensitivity RDTs was mentioned to benefit effective active case surveillance in the endemic areas. To implement the strategies, the respondent specified two training sessions for local health workers are required: testing with high-sensitivity RDT and testing with microscopy. At the national level, genome sequencing was said to be essential for national staff to perform surveillance of anti-malarial drug efficacy to address malaria recrudescence as a step towards moving into the malaria elimination phase.

### Prospects for and challenges of prophylaxis

Forest goers were in general unaware of prophylaxis for malaria. Healthcare workers questioned whether this approach is permitted for people living in endemic areas (which it currently is not in Thailand). Faced with hypothetical questions, forest goers, healthcare workers and community leaders gave positive responses to the idea of taking anti-malarials to prevent malaria when in the forest. They suggested that a person should take the drugs only when they spend nights in the forest and some mentioned that the drugs could be delivered by the VMW or the village leaders because they are a trusted and known member of the community who also provide other prevention measures. Some respondents felt that a medical doctor is a more reliable care provider to prescribe the medicine. A few suggested that forest goers may not want to travel to the hospital or malaria clinic to obtain the medicines. One respondent described preferring preventive tablets to vaccination, perceiving tablets to be immediate (more hands-on, ready-to-take) and thus more effective than a perceived uncertain long-term result of a vaccine.

*I: What do you think about taking medicine to protect from malaria?*

*R: Is there one? If there is one it would be nice. Who wouldn’t be scared of malaria?*

IDI with male, 53-year-old forest goer from Kor village, SSK.

*I: Would you be concerned?*

*R: Not really because I would not take it long-term (continuously), only when I go to the forest … if one tablet can protect for 1–2 days, or something like that … I am not sure about that but if it can protect I would like to try.*

IDI with male, 57-year-old forest goer from Dome Pradit village, UB.

*I: Would you worry about bad effects?*

*R: If it [prophylaxis] has bad effects to the body, like stomach ache or fatigue, it would not be good. We cannot work in the forest in that condition.”*

IDI with male, 52-year-old forest goer from Huay Chan village, SSK.

*I: How often would you prefer to take it?*

*R: Depends on how long one tablet would last I think, like 4–6 h for one paracetamol. If I spend 2–3 days in the forest I could take it once a day, or something like that. Especially at night because we need to be very careful. During the day we can light coil, use repellent, and wear long-sleeved clothes to protect but I am more worried during night time because we got very tired and fell asleep after hunting.*

*IDI with male, 46-year-old forest goer from Khae Dorn village, UB*.

Although most respondents were unfamiliar with prophylaxis, a few healthcare workers and malaria staff described administration of prophylaxis in the past when malaria was highly prevalent in the villages and forested areas. Healthcare workers reported providing anti-malarials for prevention purpose to villagers who logged and hunted in the forest. Respondents described that anti-malarial as a large yellow-coloured pill. They also reported that prophylaxis used to be available at malaria clinics for about a year when malaria was prevalent during 1997–1998. One staff reported giving 4–6 tablets or more of chloroquine to at-risk villagers during the malaria testing service in the village. The respondent described giving more tablets if the villagers mentioned that they would visit the forest for a longer period and requested more tablets.

"*Back then if villagers were tested for malaria, I also gave them 6 tablets of chloroquine. They told me it could protect … but there were effects. A person who took the medicine to protect, if he had malaria it was more difficult to treat. Now it is not provided anymore. [What do you think if prophylaxis were to be provided in the future?] It depends on the medicine provided, if it does not make treating infected patients difficult, it should be ok for forest goers to take. If there are no problems afterwards, no bad consequences like that."*

IDI with healthcare worker from Kham Bak village, UB.

Healthcare workers explained that in the past, they stopped providing anti-malarials to healthy individuals because they found it more difficult to treat confirmed cases with the same anti-malarial. One respondent described that the same people they provided anti-malarials to came back from the forest with malaria. Another mentioned that his patient purchased anti-malarials (referred to as “Ya Yoong” or a mosquito pill because of the mosquito symbol on the white-coloured pill) from a local pharmacy to take before going to the forest to prevent malaria.

"*Patients did not get better when we treated them with the same medicine. They were sick, get infected still. They [malaria programme] became aware of this so they cancelled it [prophylaxis] … Back then malaria patients were as many as 30-40 cases in a month […] The programme suggested that the medicine might not work because malaria is resistant to the drug. Villagers still wanted it though when I went to provide malaria test in the village."*

IDI with healthcare worker from Buntharik district, UB.

Concerns about prophylaxis were raised mostly by healthcare workers and village leaders. They highlighted the number of doses and whether taking too many tablets could make a person’s health worse, referring to potential side effects on their body (“damaging liver, kidney or stomach”). Minimizing the drug intake was suggested and preferred by healthcare workers and village leaders.

*“I think it would be good if there is a medicine to prevent. Will there be? [Do you have any concerns?] It is one convenient option for protection … although if a person were less careful, took medicine and did not protect [with other equipment], there might be negative consequences. I don’t know the extent to which the medicine is effective.”*

IDI with community leader from Samsao village, SSK.

Provincial staff said that the intervention should take into consideration local and up-to-date malaria epidemiology. The respondent suggested forest goers might take the medicine to keep but would be unlikely to take the medicine or stop taking them when they do not feel at risk. He also described that sub-district hospital staff, namely public health officials and nurses, might be a more reliable provider; but the coverage would be lower either because some villagers would not visit the health facility or the staff might be otherwise occupied and slower to provide the service. A suggestion was made that for VMWs to be the provider of the prophylactic medicine, a standard operating procedure should be set up to ensure standardized and high quality of provision of prophylaxis in each village such as prescription, monitoring, and giving advice to forest goers. Policymakers also described this concern related to the possibility of forest goers developing drug resistance to the treatment drugs if they do not take the medicine as prescribed.

"*I think prophylaxis, as protection, if taken as prescribed it should not be a problem. But if not, like if a forest goer took the medicine only during the two weeks he went to the forest, but not the whole month? If he came back and did not continue to take the medicine, would that affect him, the disease, or any drug resistance? These are concerns … because if the person is not at risk he might not continue to take it."*

IDI with provincial policymaker.

For national programme staff, prophylaxis for forest goers was not perceived to be a priority for malaria prevention and control because patients have better access to care than in the past. They also weighed benefits against concerns about drug resistance. Several considerations were identified for the feasibility of prophylaxis: efficacy of the drug, choice of regimen, side effects (short-term and long-term), price of the drug, and ethical considerations. Respondents highlighted the importance of supporting evidence for prophylaxis as prevention therapy in other countries other than for travellers. A clinical trial was suggested to provide evidence on the drug efficacy. However, the respondent was concerned whether it will be sufficient to justify what effect(s) prophylaxis could have on malaria incidence in the context of drug resistance and on patients’ safety, such as those with chronic disease or using alcohol or substances, that unlike in clinical trials, cannot be “controlled” in real-life circumstances.

*"For Thailand getting access to care is not exceedingly difficult. When a person is sick, s/he can come to get treatment within 2–3 days, usually not longer than that. So it does not seem necessary … and there are also concerns if patients will take the prophylaxis correctly or if they are infected (during taking prophylaxis) but they do not come to get treatment. Because the symptoms might be minimal. […] if a person gets sick, s/he can get to hospital care, even a sub-district hospital, within 24 hours."*

IDI with national policymaker.

The choice of regimen was seen as important for adherence to prophylaxis. Policymakers described that the number of doses and side effects need to consider the response of end-users and healthcare workers who will administer the medicine. Respondents also suggested excluding anti-malarials used for previous and current treatment regimens to avoid introducing possible new drug resistance. In addition, price was described as a crucial factor for decision-making: malaria treatment drugs are now covered under the long-term national budget, unlike external funding of malaria services from the programme. Respondents gave an example of the use of mefloquine and atovaquone/proguanil as recommended prophylaxis for travel medicine (in the United States): mefloquine is more affordable but may have more (potential) side effects whereas atovaquone/proguanil may have fewer side effects but is more expensive for patients.

The respondents also expressed concern over possible ethical issues, describing prophylaxis as a “double-edged sword”: whether the preventive therapy would discourage people from using other protection measures in the forest when they feel that they are protected from malaria (thus they may be less careful in the forest). There were questions about whether the protection that prophylaxis offered was high enough to justify making it available among forest goers as it was perceived by medical staff as not a “life-saving drug”. The case study on HIV vaccine in Thailand in 2009 [33] was given as an example of these considerations about how much protection should a preventive intervention give to be approved [34].

## Discussion

Drawing on in-depth interviews, the findings outline an overview of forest goer behaviours and perspectives in an area of northeast Thailand including their risk of malaria infection and access to malaria prevention and treatment. They also highlight the views of local and national stakeholders on their roles in addressing forest malaria and implementing malaria interventions, including essential considerations of malaria prophylaxis as a prevention measure.

### Forest goers and their risk of malaria

As described elsewhere for neighbouring countries, the findings indicate that the forest is the site of livelihood activities for communities located in or close to forested areas [35]; forest activities, including employment in rubber plantations in the forest fringe, expose this population group to risk of malaria infection. A previous study conducted 2014 in Ubon Ratchathani highlighted the economic drivers of forest going and how they were linked to a global supply chain of luxury timber; logging rosewood trees for sale was the main reason for forest going [36]. Findings outline more diverse livelihood activities beyond logging. For these reasons, forest goers reported visiting restricted areas where hunting and logging activities are prohibited or where there is a military presence (particularly along the international border) in this study and elsewhere in Thailand [37] and Cambodia [26–28, 38].

Studies of malaria epidemiology in western and southern Thailand suggested that rubber farmers and workers were exposed to biting malaria vectors during night-time rubber tapping work [39, 40]. Risk of biting exposure, both outdoors and indoors, was also high in forested farm hut sites and in forested villages [41]. Respondents were generally aware of and concerned about malaria risk, and sought to protect themselves in the forest and forest farm. Some also described their daily lives in the forest and experiences of the disease as part of their hardship from making a living in a rural livelihood with limited socio-economic resources. Their close relationship to the forest and routine work in the rubber farms make specifying a clear exposure period for taking prophylaxis difficult. Prevention interventions including prophylaxis should target forest goers who report visiting the forest and staying overnight in the forest. Travel patterns of forest goers and contextual factors (such as agricultural productivity, crop failure, preserved areas, forest tenure) are therefore critical to understand how best to target forest goers and protect them from the remaining foci of malaria transmission in forests.

### Access to malaria prevention and treatment

Many respondents considered VMWs or MPWs as essential primary care providers for malaria. Like elsewhere in the GMS (Cambodia [27, 42–45], Lao PDR [46], Myanmar [47], and Vietnam [48]), village residents in Thailand are likely to seek testing and treatment from local health workers [49, 50]. Nevertheless, their decision to seek treatment at a malaria clinic or hospital in the district depended less on its distance from home than in other countries, likely because of better access to care compared to other communities in mountainous and remote areas, particularly in the rainy season, or the perceived cost of treatment elsewhere [51]. The illegal nature of forest visits may also complicate access to malaria testing and treatment among some forest goers who do not wish to report their trips to authorities or officials. A review of access to healthcare in Thailand has underlined concerns about disclosing personal information in healthcare settings for fear of being stigmatized from other infectious diseases such as HIV or tuberculosis, posing barriers particularly among migrant populations [52]. This highlights the need to keep malaria services available and accessible at the village level despite malaria declining. Although their roles have focused primarily on malaria, village malaria services could be further integrated into the local health system and harnessed to address other diseases in the communities.

### Malaria interventions targeting forest goers

In the GMS, several interventions have been introduced to address forest malaria, including distribution of bite prevention measures [53], active case detection by Mobile Malaria Workers [54], and mass screening and treatment by forest-malaria workers [28]. Forest goers described using various bite preventive measures including long-sleeved clothes, repellents, coils, making fire, and sleeping under LLINs and LLIHNs [55]. In this study, many respondents reported use of these prevention measures and their limitations in forest settings, particularly when they engaged in labour-intensive work and resting. A recent review highlighted that the use of such vector control measures were often sub-optimal because of the nature of forest visits and limited protection of such equipment [9]: forest goers were still susceptible to mosquito bites when engaging in forest activities or resting at potential hotspots. Provision and use of multiple prevention measures based on the understanding of their risks and preferences in addition to evidence on the efficacy of such measures, are crucial to design an appropriate, more tailored forest package for forest goers.

By targeting specific populations and areas, national programme staff highlighted that low adherence to malaria treatment and detecting malaria infection in low-transmission settings remain a challenge. The ongoing transmission among mobile and migrant populations in different parts of the country reduced the programme’s capacity to monitor cases and deliver services, particularly in highly mobile populations in western and southern Thailand, and also among migrant workers and military personnel along the border [8]. Malaria programme staff suggested that a short-term (fewer dose) treatment regimen could be administered to malaria patients in these highly mobile areas. Similar to this study, recent interviews with NMCP staff in the Asia-Pacific Region also highlighted the necessity for highly-sensitive RDTs to detect asymptomatic vivax infection, and for quantitative G6PD testing to prescribe single-dose tafenoquine [56]. Recent studies on malaria elimination and addressing vivax malaria also emphasized the need to strengthen malaria diagnostic capacity at the local level to treat (6) and prevent recrudescence of vivax cases [57] in low transmission settings.

### Prospects for prophylaxis

Several factors may underpin the acceptability and uptake of prophylaxis among forest goers. When faced with a hypothetical question, forest goers described prophylaxis as a convenient way to protect themselves against malaria. Their willingness to try out the medicine to protect themselves during forest visits suggests that prophylaxis may be acceptable if a short-term regimen. However, several concerns, including the number of tablets and the length of the regimen, were raised by forest goers and local stakeholders. Respondents were also concerned whether the medicine would have long-term effects on their health. Concern about adherence to prophylaxis was also highlighted by malaria staff. Although previous studies on the use of malaria chemoprophylaxis on the Thai-Cambodia border reported high uptake of prophylaxis among Thai soldiers [25], uptake of and adherence to prophylaxis may differ between military personnel and indigenous populations, mainly because the former are likely to be closely monitored, with the prophylaxis given as directly observed therapy. For travellers, their uptake and adherence varied based on several other factors including pill burden [58, 59], cost [60, 61], perceived risk, travel characteristics [59, 60, 62], scepticism about effectiveness [60], and side effects [63].

Local and national malaria programme staff were more sceptical of prophylaxis, because of past experiences and a lack of recent studies to provide sufficient evidence for prioritizing this intervention. Decent access to care and threats from lower efficacy of the same regimen for treatment (of the same patients) were considered to weigh against prophylaxis as a strategy to address forest malaria in Thailand. A clinical trial among migrant workers in eastern Thailand suggested that the feasibility of prophylaxis administration depended on the choice of regimen, their efficacy and the safety of users [21]. A recent review of end-user perceptions on preventive anti-malarial therapy also highlighted that the level of uptake of, and adherence to, such interventions are contingent upon trust in the providers, stakeholder engagement, and integration into broader care provision [64]. This highlights that for prophylaxis to be considered a plausible approach, strong evidence on the drug’s efficacy and its safety, appropriate choice of regimen, cost-effectiveness and risk assessment needs to be made available. Its implementation should also identify an appropriate provider and necessary training, formulate criteria to deliver prophylaxis, develop pre/post testing guideline, and engage stakeholders, particularly the communities where the implementation will take place.

Despite challenges to the malaria control programme outlined above, recent studies in Thailand suggest that with high-quality clinical management of malaria and rapid case surveillance, the programme has successfully reduced malaria incidence, and may achieve malaria elimination by its target year [5, 6]. This may explain policymakers’ views of prophylaxis, along with other considerations, as less prioritized in the context where programme’s activities are already accelerating elimination.

Table 2 summarizes the main considerations for malaria prophylaxis among forest goers as a strategy. The findings indicate that forest goers and those who visit the forest are still at an increased risk of malaria infection in low transmission settings. This highlights the importance of continued use of prevention measures and provision of malaria services by VMWs in endemic communities. Despite reductions in malaria incidence, researchers have suggested that resources and efforts should be maintained to continue malaria elimination activities in remote areas and sustain elimination commitment [65]. For Thailand, evidence for continued political and financial support was provided by the national programme aiming to achieve elimination by 2024 [66]. This indicates that VMWs and the primary healthcare system are key to the elimination strategy and that support should be made available at the local level. Further research is needed on how VMWs services could be expanded and/or integrated into the local health system to sustain access to malaria services among at-risk populations in the phase of elimination.

**Table 2:** Main implications for considerations of malaria prophylaxis as a strategy

Main policy implications	
Prophylaxis as a part of malaria intervention	1. Target forest goers who are most at-risk from their activities with a clear period of exposure as well as those who work in forest farms during the night 2. Forest goers prefer minimal numbers of tablets for a short period (only during forest visits); potential side effects are the main concern among locals 3. The intervention is less prioritized in areas with good access to care. However, some forest goers in these areas may choose not to visit (or delay visiting) a public health facility for various reasons.
Choice of regimen	4. Choice of anti-malarial regimen is a key determinant of feasibility (including its cost, efficacy, length and complexity, number of tablets, potential side effects, its safety and long-term impact on users) 5. Avoid administering anti-malarials that are currently used as first-line treatment for the target population 6. Adherence to prophylactic therapy remained a key concern in the context of multi-drug resistance
Delivery of prophylaxis and provider	7. VMW and/or sub-district hospital could be an appropriate provider to deliver prophylaxis along with diagnosis and treatment; they can also monitor and follow-up to ensure uptake of, and adherence to the prophylaxis regimen 8. Training is needed for VMWs to equip them with knowledge and supply as a reliable provider as perceived by community members
Messages about prophylaxis	9. Emphasize the importance of adhering to the prophylactic medicine 10. Encourage continuing use of other protection measures from mosquito bites and visiting public health facilities for clinical treatment when they have malaria symptoms

Similar studies conducted in Cambodia [67] and Lao PDR [68] highlighted how forest activities were the main livelihoods and source of income—from foraging to farming—for forest goers in all three countries. Most forest goers had past experience with malaria and were aware of their malaria risk in the forest but the use of mosquito-bite protection was more limited among Cambodian and Lao respondents. Malaria prophylaxis was perceived to be largely acceptable among forest goers in all three countries, including by respondents in Cambodia who took part in a prophylaxis trial. Among trial participants in Cambodia, concerns about possible side effects and pill burden were viewed as the main drawbacks. Evidence of efficacy and challenges with anti-malarial resistance were among the key considerations for its implementation in these countries. However, in Thailand, the prophylactic option was viewed as less of a priority because access to healthcare was perceived to be adequate.

## Strengths and limitations

This is the first study to use qualitative research methods to explore prospects for anti-malarial prophylaxis in Thailand, and adds to previous studies on forest malaria in northeast Thailand [36]. Using a team of four trained researchers to collect data guarded against the undue influence of a single data collector on the findings. The findings are mainly drawn from reported data and might be subject to desirability bias, however, observations and informal conversations provided additional information on the context of forest going and sensitive topics, such as logging. The interviews were conducted in central Thai and Isaan (northeastern Thai) which are the spoken languages in the forest goers’ daily lives. All forest goer respondents were male, which reflects those at highest risk for malaria and the extent to which male community members are engaged in forest activities. They were diversely drawn from a range of endemic villages along the Thailand-Laos-Cambodia border area. Nevertheless, there may be other groups of forest goers, such as those with illegal status or stigmatizing diseases, that were not included. Interviews with stakeholders were conducted with multiple levels of healthcare workers (VMWs and malaria clinic staff), community leaders (village and sub-district), and policymakers (provincial and national). No interviews were conducted with international stakeholders who may offer advice on the national malaria elimination strategy. Further research would ideally explore the perspectives of stakeholders in supranational organizations, such as the WHO, on malaria prophylaxis in the GMS.

## Conclusions

In northeast Thailand, forest goers are well aware of their (elevated) malaria risk and seek care for malaria from local health providers. Although forest goers and community members in these areas have a close relationship with the forest, they are not a homogenous group: their place and time-at-risk varied according to their activities and length of stay in the forest. Several considerations should be taken in the decision-making process on implementation of malaria prophylaxis: targeting forest goers who are at-risk with a clear period of exposure, ensuring continued use of vector control measures and adherence to prophylactic anti-malarials, and adopting an evidence-based approach to determine an appropriate regimen. Implementation research alongside any future study of prophylaxis should thus monitor uptake and adherence. Beyond addressing current intervention challenges and managing malaria incidence in ongoing transmission areas, it is crucial to keep malaria services available and accessible at the village level and provide support, especially in areas home to highly mobile populations, to service provision by VMWs and local health facilities.

## Supplementary Information

**Additional file 1.** Forest goer IDI guide.

**Additional file 2.** Healthcare worker IDI guide.

**Additional file 3.** Community leader IDI guide.

**Additional file 4.** Policymaker IDI guide.

## Data Availability

The data on which this article is based cannot be shared publicly due to confidentiality of the individuals who participated in the study. The data are available upon reasonable request to the Mahidol Oxford Tropical Medicine Research Unit Data Access Committee (datasharing@tropmedres.ac) complying with the data access policy (https://www.tropmedres.ac/units/moru-bangkok/bioethics-engagement/data-sharing/moru-tropical-network-policy-on-sharing-data-and-other-outputs) for researchers who meet the criteria for access to confidential data.

## Abbreviations

ACT: Artemisinin-based combination therapy
DVBD: Division of Vector Borne Diseases
GMS: Greater Mekong Subregion
G6PD: Glucose-6-phosphate dehydrogenase
IDI: In-depth interview
IPT: Intermittent preventive treatment
IRS: Indoor residual spraying
ITN: Insecticide-treated mosquito nets
LLIN: Long-lasting insecticidal net
LLIHN: Long-lasting insecticidal hammock net
MC: Malaria clinic
MDA: Mass drug administration
MP: Malaria post
MPW: Malaria post worker
NMCP: National malaria control programme
RDT: Rapid diagnostic test
SMC: Seasonal malaria chemoprevention
SSK: Si Sa Ket
UB: Ubon Ratchathani
VMW: Village malaria worker
